# Massive Bilateral Middle Turbinate Concha Bullosa: A Case Report

**DOI:** 10.7759/cureus.60206

**Published:** 2024-05-13

**Authors:** Abdulaziz F Alfadley, Aeshah J Almahbub, Hanadi Fatani, Yazeed Alsuliman

**Affiliations:** 1 College of Medicine, Alfaisal University, Riyadh, SAU; 2 Otolaryngology - Head and Neck Surgery, King Fahad Medical City, Riyadh, SAU; 3 Histopathology, King Fahad Medical City, Riyadh, SAU

**Keywords:** giant concha bullosa, partial turbinectomy, nasal obstruction, middle turbinate, concha bullosa

## Abstract

Concha bullosa (CB) manifests as a commonly encountered anatomical variant within the middle turbinate; nevertheless, the incidence of sinusitis attributable to CB is notably uncommon. This case represents chronic rhinosinusitis associated with an unusually massive bilateral CB reaching the floor of the nasal cavity which was treated surgically by performing functional endoscopic sinus surgery and partial middle turbinectomy. This case report aims to contribute to the existing knowledge in the field of otolaryngology by elucidating the surgical options associated with massive middle turbinate CB and ultimately improving the care provided by otolaryngology surgeons for patients with similar conditions.

## Introduction

Concha bullosa (CB) is an anatomical variation of the lateral nasal wall most commonly encountered in the middle turbinate. It refers to the hypertrophy and existence of air cells within the turbinates [1]. The incidence of middle turbinate aeration is between 13% and 53.6% [2]. CB is typically asymptomatic and is often diagnosed incidentally during radiological assessments by computed tomography (CT). However, symptomatic patients may present with nasal obstruction, headache, facial pressure, or anosmia. Medical management of CB is insufficient in symptomatic patients and surgery is the usual treatment of choice. Nonetheless, the location and degree of resection of the middle turbinate remain questionable as there is no consensus currently on the optimal surgical approach [3]. In this case study, the authors discuss the unusual occurrence of a massive bilateral middle turbinate CB, which was initially mistaken for polyps based on the endoscopic image. The condition was successfully managed through surgical intervention, in the form of partial turbinectomy due to its massive size.

## Case presentation

**Figure 1:**
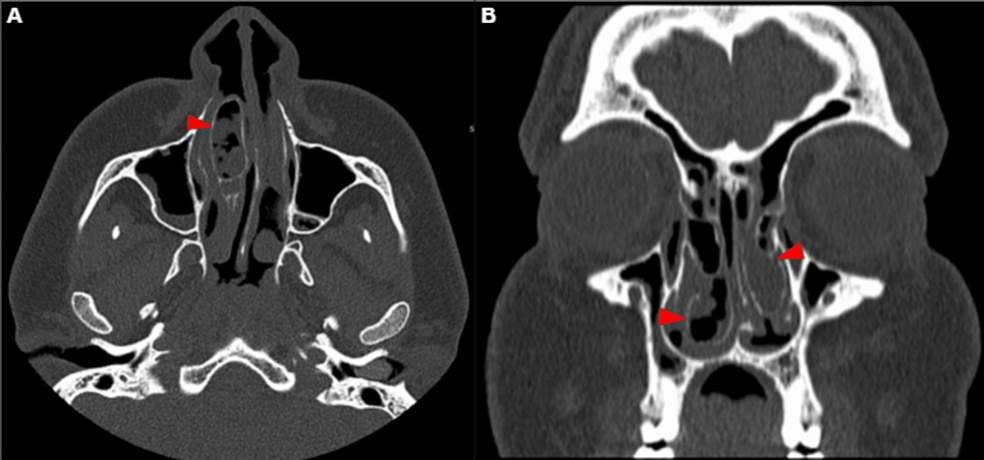
Computed tomography scan without contrast of the paranasal sinuses. Axial view (A) and coronal view (B) showing bilateral scattered mucosal thickening within the maxillary, anterior, and posterior ethmoid sinuses, bilateral middle turbinate CB filled with opacification (red arrows), and bilateral occlusion of the stomatal complexes. CB: Concha bullosa

**Figure 2:**
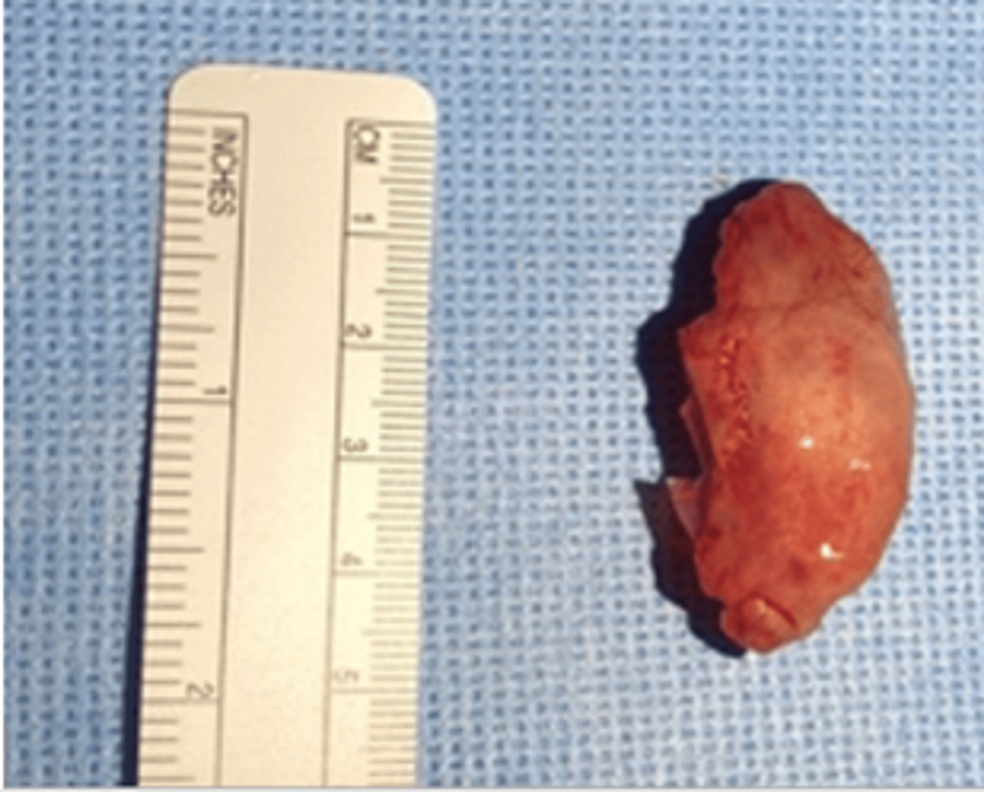
Right huge concha bullosa after middle turbinectomy reaching the size of 4.5 cm.

**Figure 3:**
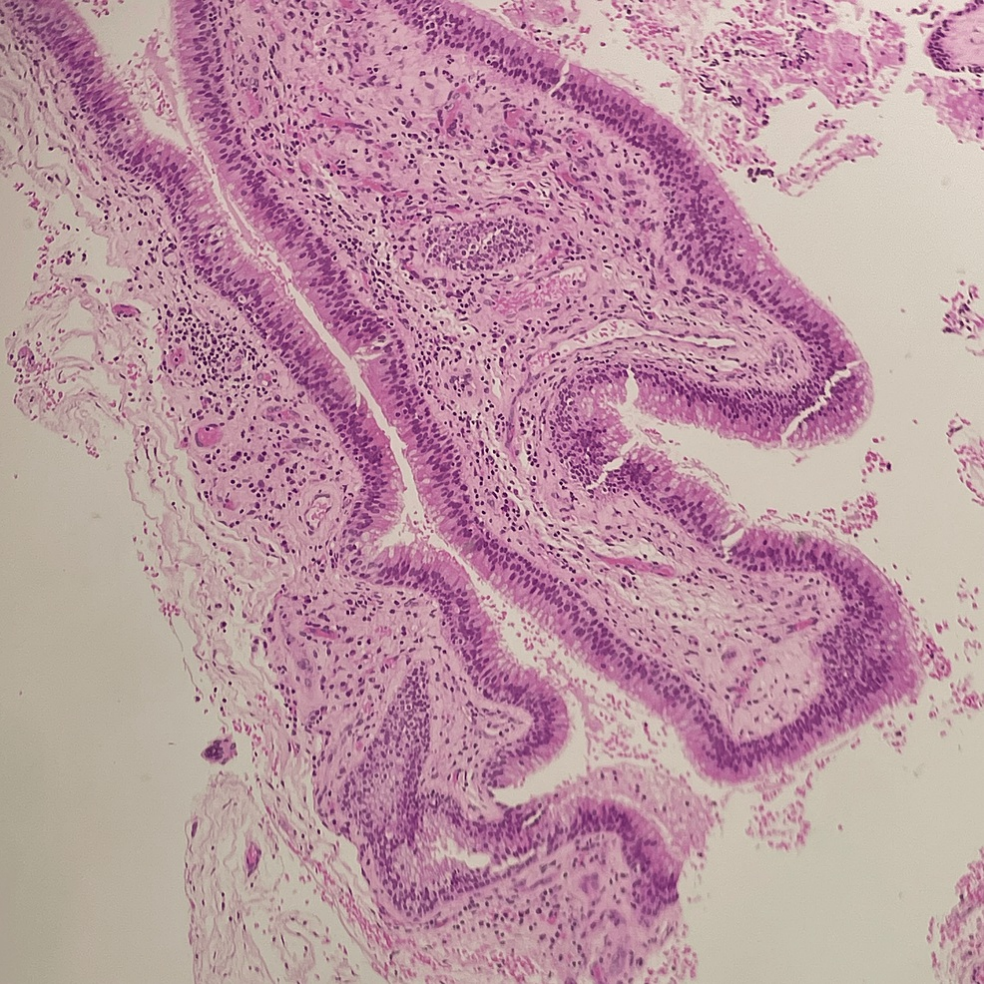
Polypoid fragments are lined by respiratory epithelium. The sub-epithelium shows mixed inflammatory cells including eosinophils.

A 19-year-old male with a known case of tetralogy of Fallot and chronic rhinosinusitis (CRS) presented to the otorhinolaryngology clinic with a history of left persistent nasal obstruction, nasal discharge, posterior nasal drip, and facial pain. On examination, nasal endoscopy revealed thick nasal discharge and complete obstruction with grade IV polyps on the left side and grade III polyps on the left based on Meltzer classification [4]. For further evaluation, a CT scan without contrast of the paranasal sinuses was performed (Figure 1). It showed significant bilateral scattered mucosal thickening within the maxillary, anterior, and posterior ethmoid sinuses, bilateral middle turbinate CB filled with opacification, and bilateral occlusion of the stomatal complexes. The patient was initially treated with appropriate CRS medical therapy which had failed. The option for surgery was offered and explained to the patient, and the risks and benefits of the procedure were discussed thoroughly. The patient underwent bilateral partial turbinectomy for the massive CB and limited functional endoscopic sinus surgery (FESS) including bilateral maxillary antrostomy and anterior and posterior ethmoidectomy with septoplasty. The specimen was collected in formalin and sent for further evaluation (Figure 2). Upon histopathological examination, a diagnosis of “sinonasal inflammatory polyp” was confirmed (Figure 3). The patient was followed up at the otorhinolaryngology clinic for one year after the surgery. He was maintained on Pulmicort irrigation and was not strictly compliant with his medication. During his follow-up visits, the patient showed improvement in all his symptoms. Nasal endoscopy in the last visit showed a patent airway bilaterally, grade 1 polyp, and mucous bilaterally with no crustation.

## Discussion

Nasal turbinates are important structures that are located at the lateral nasal wall. Normally, there are three turbinates: superior, middle, and inferior. They have a significant role in maintaining normal nasal function, which includes airflow direction regulation, humidifying, and filtering the air. Various anatomical variations of the middle turbinate have been reported in the literature [1]. It has been suggested that middle turbinate CB can play a role in the etiology of chronic sinusitis by impairing sinus ventilation and mucociliary transport [2,5]. Moreover, secondary sinusitis and frequent attacks of sinusitis in cases of giant CB have been reported [6]. According to a study conducted by Bolger et al., middle turbinate pneumatization is divided into three types based on the site of the pneumatization. The first is the pneumatization of the vertical lamella, which is called the lamellar cell. The second is pneumatization of the inferior bulbous portion of the middle turbinate, called the bulbous type. The third category was referred to as true CB, where there was extensive pneumatization of the entire middle turbinate. In the latter, both the vertical lamellar and inferior bulbous portions are involved. Among the three categories, true CB was the least frequent [7]. CB is often asymptomatic but can cause symptoms, particularly in cases of extensive pneumatization, especially if bilateral. Common symptoms include sinus-related issues like headaches due to mucosal contact, anosmia, and nasal obstruction especially if it is massively pneumatized as in our case [8,9].

Various surgical techniques have been explored in addressing CB, including lateral/medial laminectomy, total turbinectomy, partial turbinectomy, conchapexy, and crushing. Among these methods, lateral/medial partial middle turbinate surgery is the commonest procedure utilized while total turbinectomy is used the least [10]. Surgical management of massive CB is a topic that has limited literature and reported cases. According to our knowledge, there have been only four case reports discussing the presentation and surgical techniques of this entity. Out of these four reported cases, three of them involved unilateral massive CB. In all three cases, the surgical approach chosen was lateral laminectomy. All three cases had successful outcomes with no postoperative complications. This suggests that lateral laminectomy is an effective surgical technique for managing massive CB [6,10,11]. The remaining case report described a patient with unilateral giant CB and a smaller CB on the contralateral side. This case required both medial and lateral laminectomy which resulted in an improvement of all the patient’s symptoms apart from smell loss with an uneventful postoperative course [12]. However, it is important to note that none of the previous cases reported the unusual bilateral anatomical variation seen in our case. Our case involved massive CB on both sides, reaching the floor of the nasal cavity misdiagnosed as nasal polyps on clinical examination. In contrast to the previous cases, our patient was managed with bilateral partial turbinectomy with no postoperative complications and experienced complete resolution of symptoms. The follow-up duration varied among the previous studies ranging from one up to nine months. In this study, we have the data of CB postoperative course up to one year. Unfortunately, long-term outcomes beyond one year postoperatively remain limited in the literature, highlighting the need for further research in managing giant CB.

## Conclusions

This case report presents the occurrence of massive bilateral middle turbinate CB, which was initially misidentified as polyps during an endoscopic examination. The discrepancy between the clinical findings and radiological results emphasizes the challenges in diagnosing anatomical variants accurately. Therefore, a thorough review of the CT scan is crucial in the preoperative period. Partial turbinectomy and limited FESS proved to be successful, as evidenced by a significant improvement in the patient's symptoms during the follow-up period.
